# An IRF1-IRF4 Toggle-Switch Controls Tolerogenic and Immunogenic Transcriptional Programming in Human Langerhans Cells

**DOI:** 10.3389/fimmu.2021.665312

**Published:** 2021-06-15

**Authors:** James Davies, Andres F. Vallejo, Sofia Sirvent, Gemma Porter, Kalum Clayton, Yamkela Qumbelo, Patrick Stumpf, Jonathan West, Clive M. Gray, Nyaradzo T. L. Chigorimbo-Murefu, Ben MacArthur, Marta E. Polak

**Affiliations:** ^1^ Clinical and Experimental Sciences, Sir Henry Wellcome Laboratories, Faculty of Medicine, University of Southampton, Southampton, United Kingdom; ^2^ Division of Immunology, Institute of Infectious Disease and Molecular Medicine, Department of Pathology, University of Cape Town, Cape Town, South Africa; ^3^ Human Development and Health, Faculty of Medicine, University of Southampton, Southampton, United Kingdom; ^4^ Cancer Sciences, Faculty of Medicine, University of Southampton, Southampton, United Kingdom; ^5^ Institute for Life Sciences, University of Southampton, Southampton, United Kingdom

**Keywords:** Langerhans cells, single cell transcriptomics, gene regulatory network, mathematical modelling, adaptive immunity, immunotolerance, immunogenic, transcriptional programming

## Abstract

Langerhans cells (LCs) reside in the epidermis as a dense network of immune system sentinels, coordinating both immunogenic and tolerogenic immune responses. To determine molecular switches directing induction of LC immune activation, we performed mathematical modelling of gene regulatory networks identified by single cell RNA sequencing of LCs exposed to TNF-alpha, a key pro-inflammatory signal produced by the skin. Our approach delineated three programmes of LC phenotypic activation (immunogenic, tolerogenic or ambivalent), and confirmed that TNF-alpha enhanced LC immunogenic programming. Through regulon analysis followed by mutual information modelling, we identified IRF1 as the key transcription factor for the regulation of immunogenicity in LCs. Application of a mathematical toggle switch model, coupling IRF1 with tolerance-inducing transcription factors, determined the key set of transcription factors regulating the switch between tolerance and immunogenicity, and correctly predicted LC behaviour in LCs derived from different body sites. Our findings provide a mechanistic explanation of how combinatorial interactions between different transcription factors can coordinate specific transcriptional programmes in human LCs, interpreting the microenvironmental context of the local tissue microenvironments.

## Introduction

Langerhans cells (LCs), identifiable by high CD207/Langerin and CD1a expression act as immune sentinels at the epidermis and, through antigen presenting function, are responsible for maintaining tissue immune homeostasis (1). In the steady-state, a network of LCs resides within the dense assembly of epidermal keratinocytes (KCs), sensing the environment and capturing antigens through intercellular extension and retraction of dendritic processes (2). On encounter with antigen, LCs cease to phagocytose and instead upregulate pathways associated with maturation, including MHC II antigen presentation, T cell co-stimulation and migration to local lymph nodes for priming of T cell immunity (3). The plasticity of migrated LC to induce both immunogenic and tolerogenic adaptive T cell responses (4–9) has revealed the complexity in discerning the decision-making process of LCs to drive either immunogenic or tolerogenic responses and has highlighted the question as to how LCs skew T cell activation to favour responses that are preferential in different biological contexts, such as inflammation. In the context of diverse signalling from the external environment and epidermal microenvironment, LCs can promote immunogenic responses to protect against harmful pathogens, or promote tolerogenic responses to prevent unwarranted inflammation to self-antigen and innocuous agents (5, 10–12). The correct orchestration of immunogenic vs tolerogenic responses by LCs to the different stimuli they encounter is therefore expected to be fundamental to the maintenance of skin health. However, the molecular mechanisms for this decision-making process are largely unknown.

Recent investigations by us and others characterised plasticity in LC-driven adaptive immune responses, dependent on LC activation state and signalling from the skin microenvironment. In the absence of inflammation, migratory LC are marked with enhanced expression of immunocompetency genes and they preferentially promote induction of Th2 CD4+ T cell responses (5–8) and tolerogenic FOXP3+ Treg responses (9, 13, 14). In contrast, with TNF-alpha signalling, LC immunogenicity is enhanced (15). TNF-alpha is a skin proinflammatory cytokine, which is produced by epidermal KCs, as well as dermal DCs, plasmacytoid DCs (pDCs) and NK cells (16–18) in response to immunogenic stimuli. TNF-alpha stimulation of migratory LC heightens their ability to drive CD8 T cell activity through antigen cross-presentation (5–7). Consistent with enhanced T cell activation, inflammatory mediators TNF-alpha and IL-1-beta promote the upregulation of costimulatory molecules and maturation markers in LC, as well as promoting migration (19–23). Furthermore, TNF-alpha signalling augments LC mediated anti-viral immunity to human immunodeficiency virus (HIV), Influenza and Epstein-Barr virus (EBV) antigen (10, 21).

Immune cell function and changes in behaviour, such as the ones observed for LCs, are encoded by unique transcriptomic expression profiles – transcriptional programmes (5, 24–26). These transcriptional programmes are coordinated by gene regulatory networks (GRNs) in which transcription factors (TFs) cooperate to define a specific, signal-induced immune outcome (27, 28). Importantly, interactions with the external environment, tissue status (health or disease) or local microenvironmental signalling, can directly regulate the behaviour of GRN, alter transcriptional programmes and induce functional changes in cells.

Thus, we hypothesised that the decision-making process of LC-driven immunity is determined by the context of the signalling environment, through alteration of transcriptional programmes underpinning LC activation. We assumed that, while spontaneous migration in the absence of pro-inflammatory signalling reflects the scenario in which LCs mediate peripheral immune homeostasis, TNF-alpha signalling favours immunogenicity. We sought to identify specific TFs defining immunogenic and tolerogenic programmes in LCs and to determine the regulatory interactions between the phenotype-defining TFs. Combining single cell transcriptome analyses with a published toggle switch ordinary differential equation (ODE) model defining two divergent sets of TF expression, containing self-amplification and mutual inhibition (29), we identified regulatory modules defining immunogenic (*IRF1, IRF4*) and tolerogenic (*IRF4, RELB, ELK1, KRAS, SOX4*) LC phenotypes. The model was used to predict LC transcriptional programmes across abdominal skin, breast skin and foreskin-derived migrated LC, and provides a mechanistic explanation of how combinatorial interactions between different transcription factors can coordinate tissue and activation-specific transcriptional programmes in human LCs.

## Methods

### Human LC Isolation

Human skin abdominoplasty and breast skin samples were collected with written consent from donors with approval by the South East Coast - Brighton & Sussex Research Ethics Committee in adherence to Helsinki Guidelines [ethical approvals: REC approval: 16/LO/0999). Fat and lower dermis was cut away and discarded before dispase (2 U/ml, Gibco, UK, 20h, +4°C) digestion. Foreskin tissue was collected from the Medical Male Circumcision HIV prevention programme in Cape Town, South Africa. Tissue was collected with consent and approved by the University of Cape Town [ethics approvals HREC: 566/2012]. Inner and outer foreskin was dissected and processed in an identical manner to the abdominoplasty samples. Migrated LCs were extracted from epidermal explant sheets cultured in media (RPMI, Gibco, UK, 5% FBS, Invitrogen, UK, 100 IU/ml penicillin and 100 mg/ml streptomycin, Sigma, UK) for 48 hours. LC were purified through fluorescence-activated cell sorting (FACS). TNF-alpha stimulated LCs were incubated for 24 hours with 25ng/ml TNF-alpha post migration out of epidermal tissue. Antibodies used for cell staining were pre-titrated and used at optimal concentrations. A FACS Aria flow cytometer (Becton Dickinson, USA) and FlowJo software was used for analysis. For FACS purification LCs were stained for CD207 (anti-CD207 PeVio700), CD1a (anti-CD1a VioBlue) and HLA-DR (anti-HLA-DR Viogreen, Miltenyi Biotech, UK).

### Drop-seq

After FACS purification, single LCs were co-encapsulated with primer coated barcoded Bead SeqB (Chemgenes, USA) within 1 nL droplets (Drop-seq). Drop-seq microfluidic devices according to the design of Macosko et al. were fabricated by soft lithography, oxygen plasma bonded to glass slides and functionalised with fluorinated silane (1% (v/v) *trichloro*(1H,1H,2H,2H-*perfluorooctyl*) silane in HFE-7500 carrier oil). Open instrumentation syringe pumps and microscopes (see dropletkitchen.github.io) were used to generate and observe droplets, using conditions and concentrations according to the Drop-seq protocol. LCs were converted into ‘STAMPs’ for PCR library amplification (High Sensitivity DNA Assay, Agilent Bioanalyser) and tagmentation (Nextera XT, Illumina, UK). Sequencing of libraries was executed using NextSeq on a paired end run (1.5x10E5 reads for maximal coverage) at the Wessex Investigational Sciences Hub laboratory, University of Southampton.

### Transcriptomic Data Analysis

The Drop-seq protocol from the McCarrol lab was followed for converting sequencer output into gene expression data. The bcl2fastq tool from Illumina was used to demultiplex files, remove UMIs from reads and deduce captured transcript reads. Reads were then aligned to human hg19 reference genome using STAR. Analyses was performed using the python-based Scanpy pipeline(version 1.5.0) (30). High quality barcodes, discriminated from background RNA barcodes, were selected based on the overall UMI distribution using EmptyDrops (31). Low quality cells, with a high fraction of counts from mitochondrial genes (20% or more) indicating stressed or dying cells were removed. In addition, genes with expression detected in <10 cells were excluded. Datasets were normalised using scran, using rpy2 within python (32). Highly variable genes (top 2000) were selected using distribution criteria: min_mean=0, max_mean=4, min_disp=0.1. A single-cell neighbourhood graph was computed on the first principal components that sufficiently explain the variation in the data using 10 nearest neighbours. Uniform Manifold Approximation and Projection (UMAP) was performed for dimensionality reduction. The Leiden algorithm (33) was used to identify clusters within cell populations (Leiden r = 0.5, n_pcs=30). Differentially expressed genes (DEGs) between populations were identified using MAST (FDR corrected p-value<0.01, logFC>1). Cluster marker genes were identified using log regression within Scanpy. Gene ontology analysis was performed using Toppgene (FDR corrected p-value<0.05), describing biological pathways associated with gene lists. Z-scores for tolerogenic and immunogenic gene signatures were calculated for each single LC. Tolerogenic signature was composed of genes identified to be associated with DC tolerogenic function and previously shown to be enriched in tolerogenic migrated LC (9). The immunogenic signature was composed of 0-24 hour TNF-alpha stimulated LC upregulated DEGs, identified from bulk RNA-seq data (5). Regulatory network inference analysis was performed using single-cell regulatory network inference and clustering (SCENIC) within python (34).

### Directional PIDC

Notebooks from Chan et al. were adapted for the analysis and run using Julia V 1.0.5 in Jupyter Notebook. Directional network inference of *IRF1* with TNF-alpha stimulated LC upregulated DEGs was performed using PIDC algorithm (35) using scran-normalised expression data. Inference of unstimulated and TNF-alpha stimulated migrated LC TF -> target networks was performed using scran-normalised expression data of core LC TFs (9), plus *IRF1* in TNF-alpha stimulated LC and the upregulated DEGs for unstimulated and TNF-alpha stimulated LCs, respectively. Edge weights were exported, and sorted to include only transcription factors as targets. Hierarchical network was visualised using yED.

### Mathematical Modelling

The toggle-switch ODE model was adapted from Huang et al. (29), in which the observed functional interactions are depicted in an ‘influence’ network, rather than molecular mechanisms of interaction. The model is constructed from two first order ODEs which govern changes in immunogenic (*I)* and tolerogenic (*T*) programmes respectively (**Figure 3A**). Each ODE is composed of 3 terms, with the regulatory influences modelled using Hill functions to describe sigmoidal associations. The first term describes auto-amplification of each programme; the second term describes the cross inhibition between opposing programmes; the final term allows for programme decay at a constant rate. The model therefore assumes that the regulatory programmes that define each programme auto-amplify their own expression, whilst inhibiting the expression of the opposite programme.

To make a more parsimonious model we assumed that the parameters that characterise generic interactions are constant (i.e. *a*,*b,k*=1, n=4 and θ=0.5) in accordance with these parameters creating a stable attractor landscape containing 3 states as described in (Huang et al.). The tri-stable model describes a phenotypic ‘attractor landscape’ in which LCs can fall into an immunogenic (A), a tolerogenic (B) or an ambivalent (C, equal ability to stimulate tolerogenic and immunogenic responses) state (**Figure 3B**). In the phase portrait, (A) and (B) therefore represent states in which the expression of TFs from either programme is dominant over the other, whilst (C) represents a state in which there is balanced expression of both immunogenic and tolerogenic programmes. The model can therefore be utilised on single cell data to predict the phenotypic state of individual LCs by plotting single LC trajectories in state space using single cell expression data z-scores of phenotype-defining TFs.

Analysis and plotting of the ODE model was performed within MATLAB (Mathworks, Inc.). Trajectories were found using the ode45 solver and phase portraits were produced using the quiver command. TF expression values or z-scores representing expression of multiple TFs in each single cell were exported from Scanpy scRNA-seq analysis, scaled to fit phase portrait boundaries and then utilised as time 0 starting points from which trajectories were calculated and plotted. The total number of cells trajectories ending at each of the 3 attractors after simulation was quantified and then plotted as pie charts in GraphPad Prism 8 software for comparison.

## Results

### TNF-alpha Enhances Immunogenic Transcriptional Programming in Migratory LC

**Figure 1:**
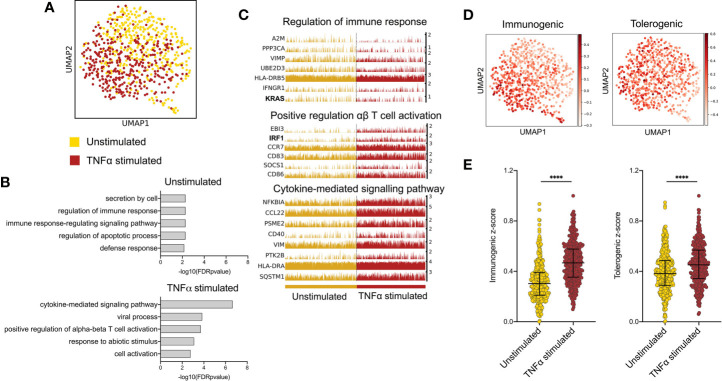
TNF-alpha enhances immunogenic transcriptional programming in migratory LC. **(A)** UMAP dimensionality reduction analysis of scran-normalised single cell data from unstimulated (375) and TNF-alpha stimulated (362) migrated LCs originating from the same donor. **(B)** Gene ontology analysis (Toppgene) for the 61 upregulated DEGs in unstimulated LCs and 87 upregulated DEGs in TNF-alpha stimulated LCs (FDR corrected p=<0.05). **(C)** Trackplots displaying genes included in ontologies upregulated in unstimulated LC (regulation of immune response) and TNF-alpha stimulated LC (positive regulation of αβ T cell activation and cytokine- mediated signalling pathway). **(D)** UMAP marker plots displaying immunogenic z-scores and tolerogenic signature z-scores in individual LC. Immunogenic z-scores were derived from the expression of genes identified to be upregulated in TNF-alpha stimulated LC (0hr-24hr DEGs) from bulk RNA-seq data (5). Tolerogenic signature z-scores were derived from the expression of genes associated with dendritic cell tolerogenic function (9). **(E)** Immunogenic and tolerogenic z-score values are displayed for unstimulated and TNF-alpha stimulated LC. Mann-Whitney test, ****p<0.001.

To investigate transcriptional programmes induced by epidermal pro-inflammatory cytokines in LCs, we performed single cell analysis of human primary migrated LCs exposed to 24h stimulation with TNF-alpha vs unstimulated control. Clustering and dimensionality reduction analysis of 775 cells (UMAP, ScanPy, version=1.5.0) revealed that LC migrated from abdominal skin and cultured in the presence or absence of TNF-alpha contained a predominant large cluster, confirmed to be LCs through high expression of MHC II genes (CD74, *HLA-DRB1, HLA-DRB5*), as well as two additional populations identified to be melanocytes (TYRP1, TYR) and T cells (CD3D) (Logistic regression, ScanPy pipeline, version=1.5.0), which were removed from downstream analysis (**Supplementary Figures 1A–C**). The heterogeneity of the 737 LCs cultured with or without TNF-alpha (unstimulated = 375, TNF-alpha stimulated = 362) was then analysed. Overall, the cells appeared relatively homogeneous, consisting of one overall large population of LCs comprising sub clusters of unstimulated and TNF-alpha stimulated LCs, which appear to diverge away from each other (**Figure 1A**). Differentially expressed gene (DEG) analysis comparing LCs with and without TNF-alpha identified 61 genes upregulated in unstimulated LCs and 87 genes upregulated in TNF-alpha stimulated LCs (MAST, adj.p-value<0.05, **Supplementary Figure 1D**). Gene ontology analysis of the 61 genes upregulated in unstimulated LCs showed they were associated with secretion by cell (adj. P-Value=5.3E-3) and regulation of the immune response (adj. P-Value=5.3E-3, **Figures 1B, C**), with the latter ontology including the TF *KRAS* (**Figure 1B** and **Supplementary Figure 1E**). Gene ontology analysis for the 87 genes upregulated in migrated TNF-alpha stimulated LCs revealed association with cytokine-mediated signalling pathways (adj. P-Value=2.2E-7) and positive regulation of alpha-beta T cell activation (adj. P-Value=1.5E-4), with the TF *IRF1* included in the ontology of the latter (**Figures 1B, C**). Unbiased clustering analysis (leiden r=0.5) identified clusters defined by unique gene expression (**Supplementary Figure 1F**). Cluster 1, which localised where mostly TNF-alpha stimulated LC were positioned on the UMAP were associated with inflammatory processes (alpha-beta T cell activation, adj. P-Value=4.4E-3), whilst Cluster 0, which localised where mostly unstimulated LC were positioned were associated with negative regulation of the immune system process (adj. P-Value=2.6E-3, **Supplementary Figure 1G**). Comparison between z-score enrichment values of immunogenic and tolerogenic LC gene expression programmes (**Figure 1D** and **Supplementary Table 1**) revealed that TNF-alpha stimulated LCs displayed a substantial enhancement for the immunogenic signature (Median = 0.4669) compared to unstimulated (Median = 0.3033, Fold change = 1.55, Mann-Whitney test, p=<0.001). A more moderate enhancement for the tolerogenic signature was observe between TNF-alpha stimulated (Median = 0.4519) and unstimulated LCs (Median = 0.3832, Fold change=1.18, Mann-Whitney test, p=<0.001) (**Figure 1E**).

### *IRF1* Expression Controls Immunogenic Transcriptional Programming

**Figure 2:**
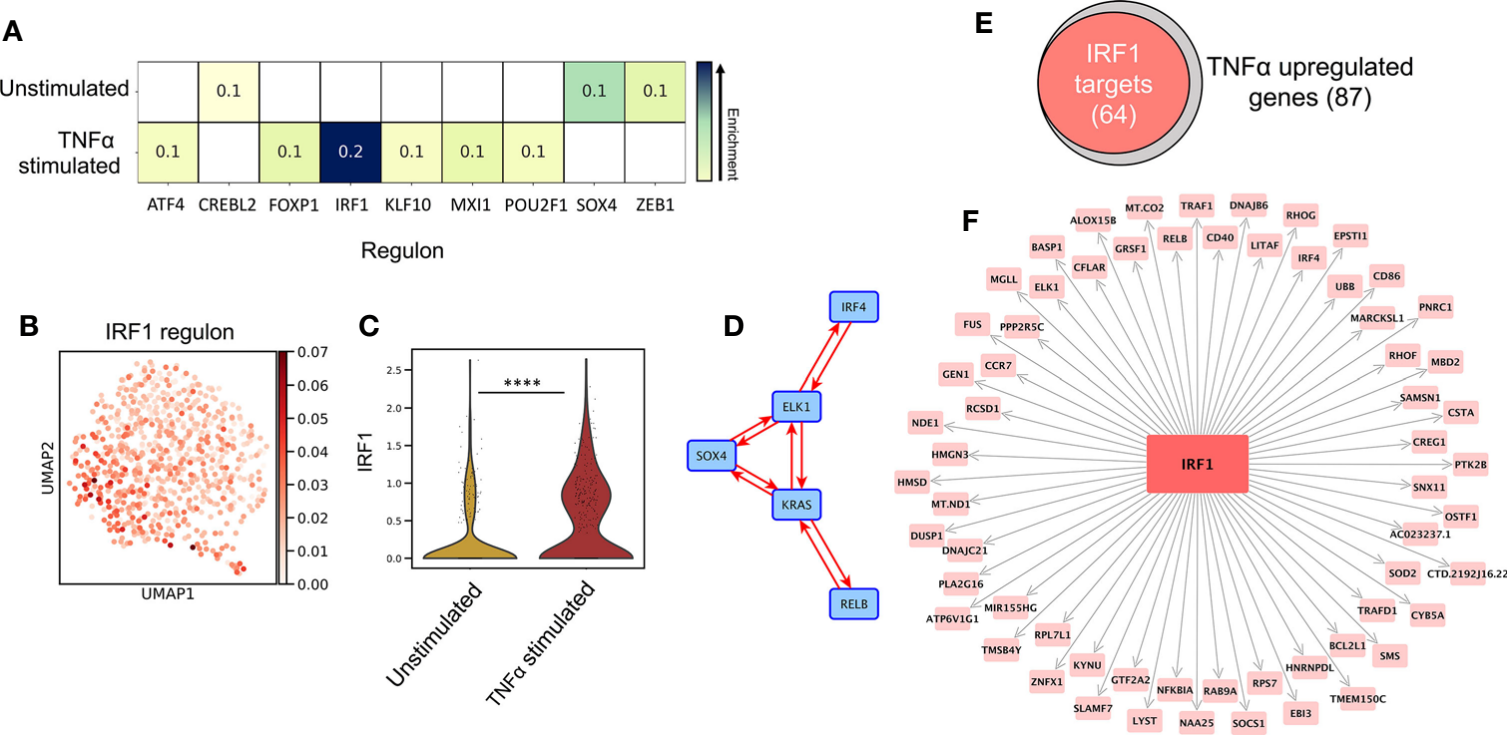
IRF1 expression controls immunogenic transcriptional programming. **(A)** SCENIC regulatory network and inference clustering analysis revealed TF regulons which were enriched in unstimulated and TNF-alpha stimulated LCs. Z-score heatmap (yellow -> blue) of enriched regulons are displayed (z-score>0.1). **(B)** UMAP marker plot displaying *IRF1* regulon enrichment (z-score) in individual LCs. **(C)** Violin plot displaying the level of transcriptomic expression of *IRF1* in unstimulated and TNF-alpha stimulated LCs, MAST, ****p<0.001. **(D)** PIDC network graph displaying connectivity (edge weight >1.5) between a regulatory module comprising of SOX4, KRAS, IRF4, RELB and ELK1 in unstimulated LCs. **(E)** Venn Diagram displaying the overlap in TNF-alpha stimulated LC upregulated genes identified to be targets of *IRF1* in PIDC analysis (edge weight >1). **(F)** PIDC network graph displaying IRF1 targets (edge weight >1) identified within TNF-alpha stimulated LC upregulated genes.

SCENIC (34) single cell regulatory network inference analysis identified the key TF regulators of programming in unstimulated vs TNF-alpha-stimulated LC, (**Figure 2A**, z-score enrichment ≥0.1). Here, TNF-alpha-stimulated LC displayed enrichment of the *IRF1* regulon (**Figure 2B**, z-score=0.2), which, along with the upregulated expression of *IRF1* from DEG analysis (**Figure 2C**, MAST), strongly highlighted this TF as being a candidate critical for immunogenic LC programming. In unstimulated LC, the most enriched regulon was *SOX4*, although this enrichment was more moderate (**Figure 2A**, z-score=0.1). Interestingly, *IRF4*, which has been demonstrated to be critical for both LC immunocompetent and tolerogenic programming (5, 9), displayed homogenous regulon enhancement and expression across both populations (**Supplementary Figures 2A, B**).

Whilst unstimulated LCs displayed significant upregulation of *KRAS* and enrichment of the *SOX4* regulon, these findings were relatively weak and less exclusive to unstimulated LC in contrast to the clear upregulation of *IRF1* in TNF-alpha stimulated LC (**Figures 2B, C** and **Supplementary Figure 2B**). We therefore explored whether these TFs acted in accordance with core TF mediators of programming in LC which have previously been associated with coordinating immunocompetent and tolerogenic regulation, including *IRF4, RELB, ELK1, KLF6* and *HMGN3* (9). Using partial information decomposition analysis (PIDC) (35) gene regulatory network inference of the 61 genes upregulated in unstimulated LC, along with *KRAS, SOX4, IRF4, RELB, ELK1, KLF6* and *HMGN3*, a directed PIDC (TF -> target gene edges only) network graph depicting regulatory interactions between TFs and target genes was generated (correlation score >1.5). Here, *KRAS* and *SOX4* could be observed to be components of a highly interconnected regulatory hub with *IRF4, RELB* and *ELK1* (**Figure 2D**). This regulatory hub could be associated with controlling the expression of 33 unstimulated LC upregulated genes (**Supplementary Figure 2C**). PIDC analysis was also performed to identify targets of *IRF1* within the TNF-alpha-upregulated gene list to discern the TFs influence on the transcriptomic programming on TNF-alpha-stimulated LC. Here, 64/87 (74%) TNF-alpha-upregulated genes were identified to be targets of *IRF1* (**Figures 2E, F**). Furthermore, PIDC analysis of *IRF1* along with the core migrated LC TFs and the 87 genes upregulated in TNF-alpha stimulated LC, suggested *IRF1* upregulation added an additional layer of regulation beneath the core network of *ELK1, RELB, IRF4* and *HMGN3* to mediate immunostimulatory programming (**Supplementary Figure 2D**).

### A Toggle Switch Mathematical Model Predicts Immunostimulatory vs Tolerogenic LC Phenotypes From Single Cell Transcriptomic Data

**Figure 3:**
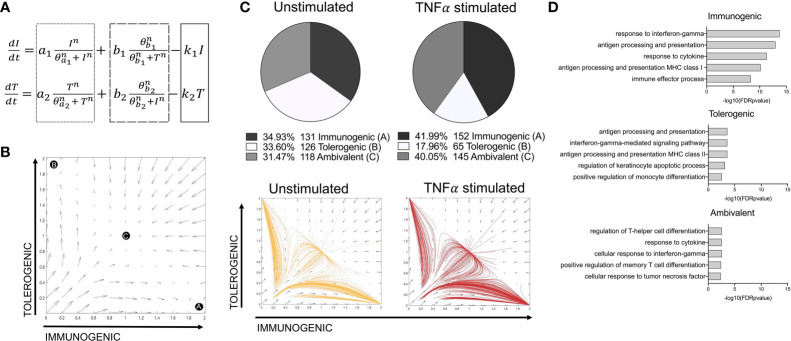
A toggle switch mathematical model predicts immunogenic vs tolerogenic LC phenotypes from single cell transcriptomic data. **(A)** Dynamical system representing the activation of immunogenic (I) and tolerogenic (T) programmes in LCs. The dotted box represents terms describing the auto-amplification of each respective programme. The dashed box represents terms describing the cross-inhibition from opposing programmes, whilst the solid box depicts the first-order decay rate (k) for each programme. **(B)** Phase portrait of the toggle switch model in which the two programmes (immunogenic and tolerogenic) auto-amplify their own expression and are mutually repressive. Black circles (A, B and C) represent end points for trajectories at stable attractors representing an immunogenic programme **(A)**, tolerogenic programme **(B)** or an ambivalent programme **(C)**. **(C)** Pie charts summarising the numbers and percentages of LC assigned to each phenotype through utilising the toggle-switch model for trajectory plotting. For each trajectory, representing an individual unstimulated or TNF-alpha-stimulated LC, the x-axis represents z-scores combining normalised *IRF1/IRF4* expression values; the y-axis represents the z-scores combining *SOX4, KRAS, IRF4*, *RELB* and *ELK1* expression values. Z-scores were scaled to fit phase portrait boundaries. **(D)** Gene ontology analysis (Toppgene) for top 100 genes expressed by immunogenic, tolerogenic and ambivalent LC (FDR corrected p=<0.05).

A tri-stable toggle switch ODE model (29) in which different activation programmes can be described based on the expression of a selected number of programme defining TFs (immunogenic vs tolerogenic, **Figures 3A, B**) offered explanation on how the balance of LC immunogenic and tolerogenic phenotypes is controlled. The model has been systematically tested by iterative application of distinct transcription factor combinations (**Supplementary Table 2**). For defining the immunogenic phenotype, *IRF1* alone or in combination with *IRF4* was tested. The inclusion of *IRF4* for immunogenic regulation was based on previous analysis demonstrating the importance of *IRF4* for both immunizing and tolerizing T cell activation, as well as immunocompetent LC programming (5, 36), which was supported by our PIDC analysis which revealed extensive interconnectivity of these TFs. For defining the tolerogenic phenotype, combinations of *KRAS, SOX4, IRF4, RELB* and *ELK1* were investigated. Overall, many model iterations depicted the observations that the TNF-alpha-stimulated LC population contain increased quantities of immunogenic LCs (**Supplementary Table 2**). However, model 14, in which both *IRF1* and *IRF4* depicted the immunogenic phenotype and *KRAS, SOX4, IRF4, RELB* and *ELK1* depicted the tolerogenic phenotype, was best at predicting results in line with both criteria (**Figure 3C** and **Supplementary Table 2**). Here, the relative quantities of immunogenic (34.93%), tolerogenic (33.60%) and ambivalent (31.47%) LCs in unstimulated LCs was equal, whilst TNF-alpha stimulated LCs displayed an increase in immunogenic (41.99%) and ambivalent (40.05%) programmed LCs and a decrease in tolerogenic (17.96%) LCs. When the most polarising clusters (Clusters 1 and 2) identified by unbiased clustering analysis (**Supplementary Figures 1F, G**) were analysed with the model, the relative quantities of immunogenic (30.40%), tolerogenic (29.67%) and ambivalent (39.93%) LCs in Cluster 2 LCs was fairly equal, whilst Cluster 1, displayed a higher number of immunogenic (46.10%) programmed LCs and a decrease in tolerogenic (18.79%) and ambivalent (35.11%) LCs (**Supplementary 3A**).

The transcriptomes of immunogenic, tolerogenic and ambivalent LCs was then investigated. Here gene ontology terms for the top 100 expressed genes in each population (**Figure 3D**) revealed that immunogenic LCs were highly enriched for genes associated with antigen processing and presentation MHC class I (adj. P-Value=1.14E-13) and the immune effector process (adj. P-Value=5.21E-9). Similar ontologies were identified for tolerogenic LC, although the enrichment was significantly lesser and they also displayed the term positive regulation of monocyte differentiation (adj. P-Value= 2.87E-03). Interestingly, ambivalent LC had high expression of genes associated with regulation of T-helper cell differentiation (adj. P-Value=2.81E-03).

### IRF1/IRF4 Toggle-Switch Determines Body-Site Specific Differences in LC Immunogenic Programming

**Figure 4:**
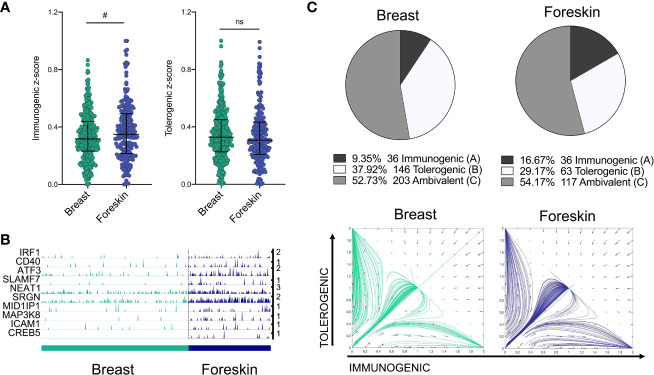
IRF1/IRF4 toggle-switch determines body-site specific differences in LC immunogenic programming. **(A)** Immunogenic and tolerogenic z-score values are displayed for.breast derived skin and foreskin LCs. Mann-Whitney test, ^#^p<0.1, ns, not significant. **(B)** Trackplots comparing the expression of transcripts associated with immunogenic LC function across breast skin derived and foreskin LCs. **(C)** Pie charts summarising the numbers and percentages of LC assigned to each phenotype through utilising the toggle-switch model for trajectory plotting. For each trajectory, representing an individual breast-derived or foreskin-derived LC, the x-axis represents z-scores combining normalised IRF1/IRF4 expression values; the y-axis represents the z-scores combining SOX4, KRAS, IRF4, RELB and ELK1 expression values. Z-scores were scaled to fit phase portrait boundaries.

We next sought to validate the power of the model to predict differences in transcriptomes from LCs of independent datasets, including a single cell dataset of migrated breast skin-derived and foreskin-derived LC. Comparative analysis of z-score enrichment for immunogenic vs tolerogenic signatures indicated dominance of immunogenic vs tolerogenic programming in foreskin LCs in comparison to LCs isolated from breast tissue. While foreskin LC more frequently display a predominant immunogenic phenotype compared to breast (**Figure 4A**, (median=0.3487) vs median=0.3171, Fold change = 1.1, Mann-Whitney test, p=<0.1), the tolerogenic signature was decreased in foreskin (median=0.3071 vs median=0.3283, Fold change = 0.94). This enhanced immunogenic programme in foreskin LCs could be seen in the expression of inflammatory pathway-associated transcripts, which importantly, included *IRF1* (**Figure 4B**). The model was then applied, using the same parameters and TFs as in **Figure 3C**, to test model predictions of immunogenic, tolerogenic and ambivalent populations amongst breast skin and foreskin-derived LC. Here the model predicted breast skin LCs to be 9.35% immunogenic, 37.92% tolerogenic and 52.73% ambivalent, whilst foreskin LCs were predicted to be 16.67% immunogenic, 29.17% tolerogenic and 54.17% ambivalent (**Figure 4C**). Model predictions of increased immunogenicity in foreskin LC therefore reflected transcriptomic observations in which foreskin derived LC display enhancement of immunogenic programming.

## Discussion

Langerhans cells (LCs), residing in the epidermis, are able to induce potent immunogenic responses and also to mediate immune tolerance. The mechanisms regulating the switch from tolerogenic to immunogenic behaviour have not been identified to date.

To address this key question underpinning homeostasis in human epidermis we analysed single cell transcriptomic data arising from unstimulated and TNF-alpha-stimulated LC, to discern the divergent programming of LCs in response to inflammatory stimuli and uncover critical TFs which govern immunogenic gene regulation.

The epidermal inflammatory cytokine TNF-alpha is a powerful mediator of inflammation and its effects on enhancing LC activation and programming of immunogenic T cells has previously been demonstrated (5, 37, 38). Here we demonstrate at the single cell level that, compared to unstimulated LCs, TNF-alpha causes divergent transcriptional programming characterised by upregulation of genes associated with inflammatory cytokine signalling processes and T cell activation, thus reflecting their enhanced immunogenic function *in vitro.* Interestingly whilst the effects of TNF-alpha were clear, there was a significant overlap between the stimulated and unstimulated populations, suggesting that common transcriptomic features, likely associated with migration and immunocompetency, were still present. Our analysis revealed that whilst both immunogenic and tolerogenic signatures were increased in TNF-alpha stimulated LC, the increase in immunogenic gene expression was significantly higher, suggesting that the overall immune response outcome is directed by the most dominantly expressed programme.

Immune cell function and behaviour are encoded by unique transcriptomic expression profiles – transcriptional programmes (24). To investigate molecular mechanisms regulating the switch from tolerogenic to immunogenic features in LCs we employed *in silico* modelling of the discovered LC transcriptional programmes. Changes in the transcriptional programmes are coordinated by gene regulatory networks (GRNs) in which transcription factors (TFs) play an essential role (27, 28). However, large scale investigations into the activity of individual GRN components and interactions between specific modules which underlie different transcriptomic programmes, and in particular the kinetics in which those programmes are executed, are difficult to investigate using functional *in vitro* methods (39). Therefore, mathematical modelling techniques are increasingly being utilised to counter this problem and include methods such as ordinary differential equation (ODE) modelling and Petri net modelling (40, 41). Mathematical modelling can permit investigations of dynamic biological systems *in silico* to assess how different molecular signals can alter regulatory network behaviour. For example, using Petri net modelling we previously documented the role of LC IRF-GRN in regulating immunogenic immune activation in response to different stimuli (10). However, Signalling Petri Net (SPN) and similar methods allow only qualitative assessment of network behaviour, and limit the strength of predictions. In contrast, ODE modelling has allowed exploration of small TF networks and specific network elements, such as positive feedback based switches, which can define cell lineage determination and operon activation (29, 42).

In GRNs, TFs act in concert with each other to coordinate different expression programmes. However, specific cellular phenotypes are determined by the increased expression of specific phenotype-defining TFs. For example, in macrophages, whilst *NFKB1, JUNB* and *CREB1* define core programmes of activation, *STAT4* is specifically upregulated in the context of chronic inflammation, which correlates with increased expression of a unique gene expression programme (24). *In vivo* analysis of LC behaviour in humans is unfeasible and *in vitro* methods to observe phenotypic behaviours are constrained. The utilisation of mathematical modelling is therefore fundamental to augmenting comprehension of phenotypic programmes of LC *in situ*. Importantly, interpretation of transcriptomic observations in light of a well-established toggle-switch model of general cell fate specification (29) permitted an unprecedented opportunity to explore the determinants regulating immunogenic vs tolerogenic programmes in LC. In our model, while expression of *IRF4* induces LC maturation and immune-competence, essential both for tolerogenic and immunogenic responses, expression of *IRF1*, induced by TNF-alpha signalling, fine-tunes the programmes towards immunogenicity. Importantly, as highlighted from our analysis, the TF *IRF1* was revealed to be a critical component of the TNF-alpha-enhanced transcriptomic programme, which appeared to be projected onto the core migrated LC transcriptional network to enhance immunogenic programming. The association of *IRF1* with inflammatory pathway activation has been observed in other systems. In DCs, TLR-9-mediated IRF1 induction leads to the induction of IFN-gamma and interferon-stimulated genes, driving efficient anti-viral immune responses (43). *IRF1* activation in macrophages is associated with the polarisation of macrophages towards the pro-inflammatory M1 phenotype (44). In fibroblast like synoviocytes (FLS), which are implicated in the inflammation in rheumatoid arthritis, TNF-alpha-mediated induction of *IRF1* leads to induction of inflammatory mediators, such as IFN-gamma (45). In contrast, *IRF4* has been conclusively demonstrated as a transcription factor critical for LC immunocompetent programming and DC capacity to induce immunogenic T cell activation (5, 36). Therefore, we hypothesised that together, *IRF1* and *IRF4* complementarily coordinate LC immunogenic programming.

Additionally, we revealed that in unstimulated LCs, *KRAS* and *SOX4* interact with components of a core network of TFs enhanced upon LC migration (*IRF4, RELB* and *ELK1*), previously demonstrated to be responsible for immunocompetent and tolerogenic regulation (9). This revealed the preference by unstimulated LC, for homeostatic regulation as compared to the immunogenic regulation enhanced in TNF-alpha-stimulated LC. Analysis of this model indicates that 3 stable phenotypes are possible, which could reflect the phenotypic landscape in which LC can adopt predominantly immunogenic or tolerogenic programmes, or an intermediate ambivalent programme, in which immunogenic and tolerogenic activation are mutually present and in balance. Such “multilineage priming” is common in cell fate switches and may have an important role in regulating LC fate decisions. To define the best model we simulated transcriptomic data *in silico* using the toggle switch models for a range of TF combinations, and compared the model predictions with observed LC status. Using the model in which *IRF1/IRF4* determine immunogenicity and *KRAS/SOX4/IRF4/RELB/ELK1* determine tolerogenicity, we demonstrated that model predictions were reflective of our transcriptomic data. Moreover, the model allowed prediction of *in vitro* phenotypic features of enhanced immunogenicity in TNF-alpha stimulated LC (5, 6).

Interestingly, the model suggested that whilst the LC population is heterogeneous, distinct populations of immunogenic, tolerogenic and ambivalent LCs could be driving unique immune responses. Further investigation into whether these states are stable, or describing continuously changing heterogeneity in response to local environmental changes, as coordinated by transcription factor expression changes, could reveal the level of influence each state has on overall LC mediated immunity.

The heterogeneity in LC population, indicating existence of a spectrum of LC immunocompetence *in situ*, can be reflected in distinct combinations of transcription factor expression by individual LC, translating into higher susceptibility of LC sub-populations to inflammatory vs tolerogenic signalling (10), and differential response to it. We hypothesised, that the expression of transcription factors modulated by the local microenvironment and could therefore be heterogenous across different anatomical sites having a localised influence on immune response outcomes. Indeed, such local differences has been reported by others for fibroblast transcriptional programmes, memory status of T cell subsets in foreskin and also for LC residing in oral vs genital mucosa (46–48).

The foreskin microenvironment is associated with increased need for effective anti-microbial responses and is reported to be a pro-inflammatory/immunologically active tissue marked by elevated pro-inflammatory cytokines and infiltration of effector immune cells (48–51). Apart from baseline and mitogen-induced TNF-alpha and IFN-gamma secretion by foreskin CD8 T cells being higher than levels secreted by CD8 T cells in the blood (48), the foreskin is most likely in a consistent state of inflammation being driven by infiltrating T cells and elevated LC’s upon exposure to a multitude of microbial stimuli (51). These inflammatory-associated characteristics of the foreskin site were reflected in transcriptomic observations made during comparison of LC derived from breast skin and foreskin, in which immunogenic programming was enhanced in foreskin LC. Furthermore, *in silico* simulations of the IRF1/IRF4 toggle switch predicted, that the LCs isolated from foreskin are likely predisposed towards immunogenic responses, highlighting the power of the model across anatomically diverse LC datasets.

In conclusion, we have shown that epidermal signalling, such as pro-inflammatory TNF-alpha, can modulate the proportion of LCs exhibiting different immunological programmes. This may therefore, reflect how LCs balance the need for different immunological responses to diverse biological stimuli. Furthermore, we have highlighted specific TF regulators critical for the modulation of both immunogenic and tolerogenic LC programmes, which, when translated into a mathematical model, have demonstrated the potential to predict LC phenotypes across different LC transcriptomic datasets. This opens opportunities to apply the model for predicting LC activation states and behaviour across different biological contexts in health and disease, and provides a tool for assessment of LC activation status in human skin.

## Data Availability Statement 

The datasets presented in this study can be found in online repositories. The names of the repository/repositories and accession number(s) can be found below: https://www.ncbi.nlm.nih.gov/geo/, GSE166079.

## Ethics Statement

The studies involving human participants were reviewed and approved by South East Coast - Brighton & Sussex Research Ethics Committee. The patients/participants provided their written informed consent to participate in this study.

## Author Contributions 

JD, MP and BM intellectually conceived and wrote the manuscript, planned the experiments and analysed the results. JD and SS performed *in vitro* experiments. JD, AV, SS, KC, PS and JW optimised and performed single cell sequencing. JD, AV and MP analysed the single cell data. BM, GP and JD optimisation and analysis of mathematical modelling. YQ and NC-M performed isolation of foreskin LC. MP, BM, NC-M and CG: discussions, data analysis, reviewing of the manuscript. All authors contributed to the article and approved the submitted version.

## Funding

The study was funded by a Sir Hendy Dale Fellowship from Wellcome Trust, 109377/Z/15/Z. Development of single cell Drop-Seq technology was funded by MRC grant MC_PC_15078. Foreskin LC isolation was funded by a South African NRF Thuthuka funding grant, TTK150624120787.

## Conflict of Interest

The authors declare that the research was conducted in the absence of any commercial or financial relationships that could be construed as a potential conflict of interest.
