# Supplementary material for: An IRF1-IRF4 Toggle-Switch Controls Tolerogenic and Immunogenic Transcriptional Programming in Human Langerhans Cells
Source: Front Immunol. 2021 Jun 15;12:665312. doi: 10.3389/fimmu.2021.665312 (PMC8239435; doi:10.3389/fimmu.2021.665312)
Supplement: Supplementary file 1 [file DataSheet_1.pdf]

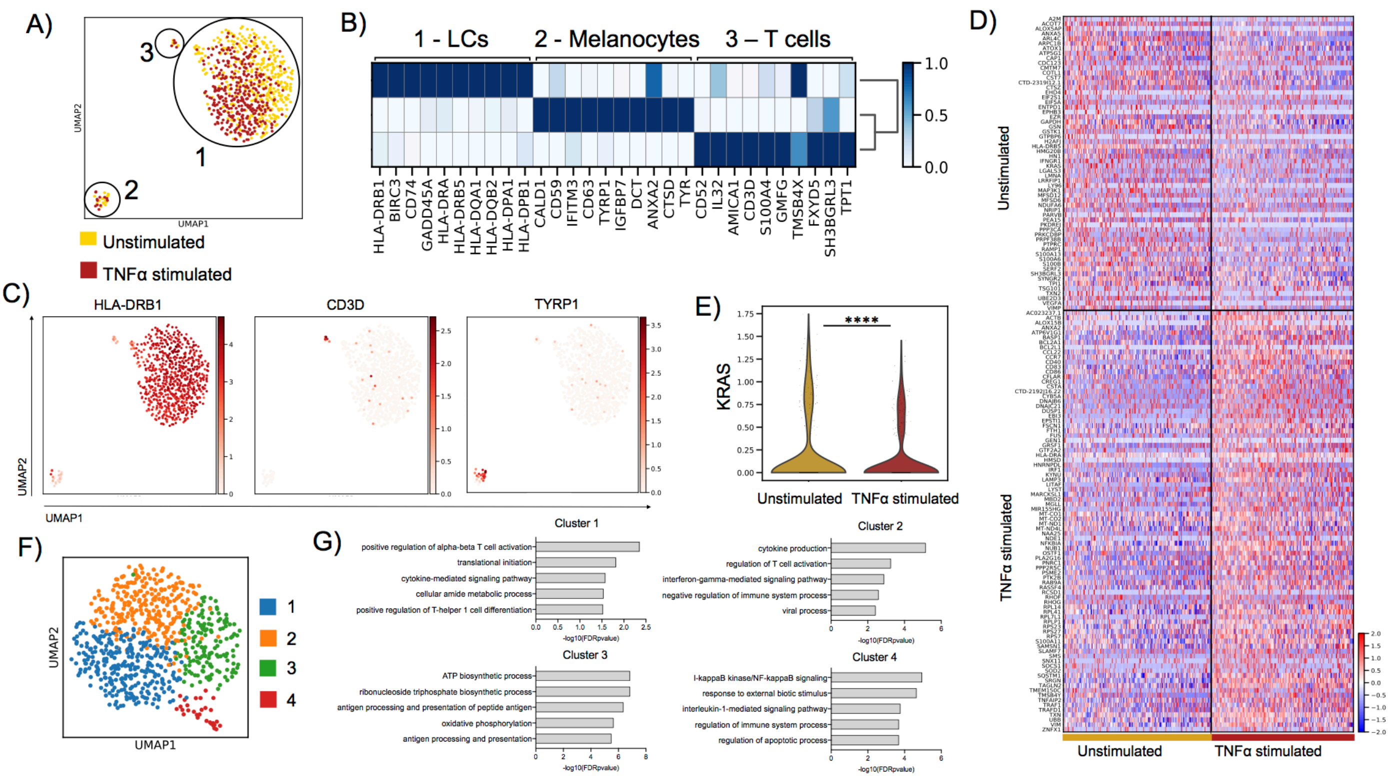

Supplementary figure 1. TNF enhances immunogenic transcriptional programming in migratory LC.

(A) UMAP dimensionality reduction analysis of epidermal cell populations detected 3 distinct subclusters of cells.

(B) Top 10 markers genes for clusters 1-3 (t-test, ScanPy pipeline, version=1.5.0), revealed populations to be LCs (cluster 1), melanocytes (cluster 2) and T cells (cluster 3)

(C) UMAP marker plots displaying the expression of the LC marker *HLA-DRB1,* the T cell marker *CD3D* and the melanocyte marker *TYRP1.*

(D) Heatmap displaying the 61 upregulated DEGs in unstimulated migrated LCs and 87 upregulated DEGs in TNF stimulated migrated LCs (FDR corrected p=<0.01, logFC>1). Gene ontology analysis (Toppgene) results are displayed alongside for unstimulated and TNF stimulated migrated LC upregulated DEGs (-log10 FDR corrected p-values)

(E) Violin plot of *KRAS* expression in unstimulated and TNFα stimulated migrated LC.

(F) UMAP plot displaying unbiased leiden clustering (r=0.5)

(G) Gene ontology analysis for the top 50 marker genes (log regression) of the clusters defined by leiden.
