# Supplementary material for: An IRF1-IRF4 Toggle-Switch Controls Tolerogenic and Immunogenic Transcriptional Programming in Human Langerhans Cells
Source: Front Immunol. 2021 Jun 15;12:665312. doi: 10.3389/fimmu.2021.665312 (PMC8239435; doi:10.3389/fimmu.2021.665312)
Supplement: Supplementary file 2 [file DataSheet_2.pdf]

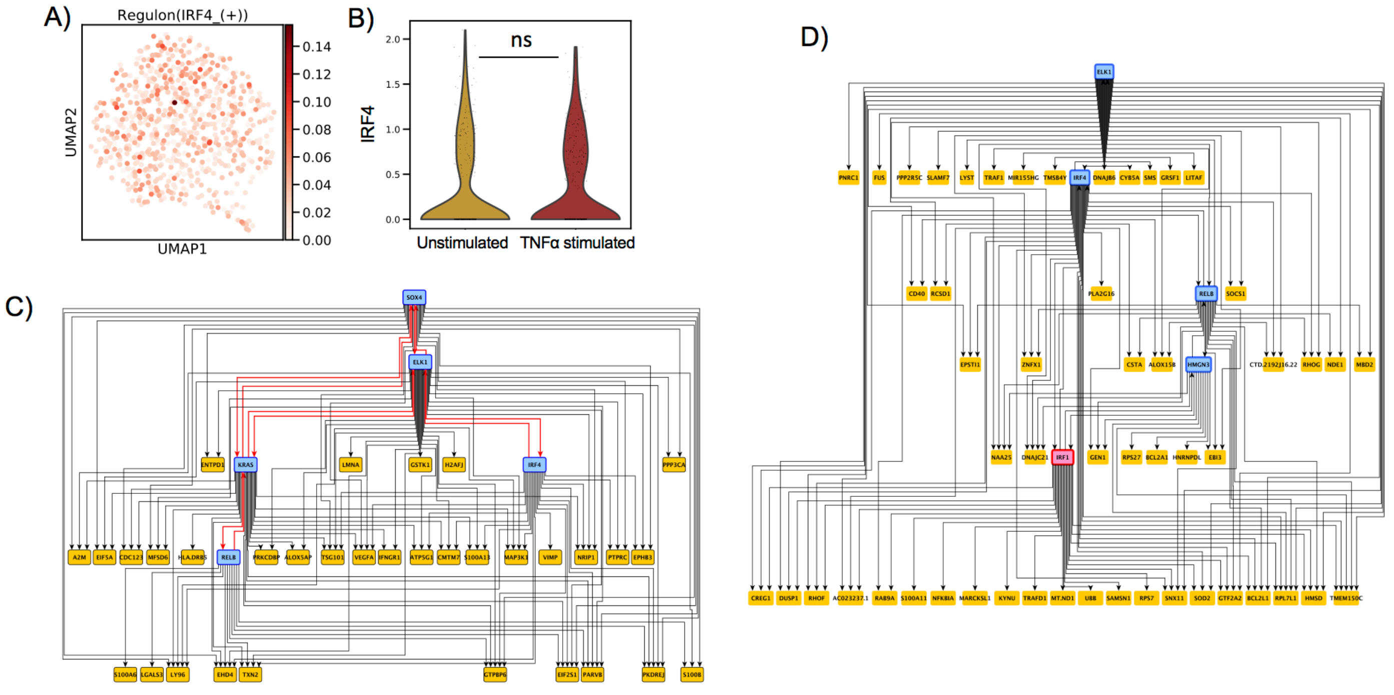

Supplementary figure 2. IRF1 expression controls immunogenic transcriptional programming.

(A) UMAP marker plot displaying *IRF4* regulon enhancement (z-scores) within individual LCs.

(B) Violin plot of *IRF4* expression in unstimulated and TNF stimulated migrated LCs. MAST, ****=p<0.001.

(C) PIDC network graph comprising 38 nodes (5 TFs, 33 output genes) and 107 edges with weight >1.5, hierarchically organized, displaying predicted regulatory modules for the regulatory TF module from Supplementary Figure 2C along with upregulated DEGs in unstimulated LCs.

(D) PIDC network graph comprising 58 nodes (5 TFs, 53 output genes) and 122 edges with weight >1.5, hierarchically organized, displaying predicted regulatory modules for *IRF1* and TFs core to migrated LC (*IRF4, HMGN3, ELK1* and *RELB*), along with upregulated DEGs in TNF stimulated migrated LCs.
