# Supplementary material for: An IRF1-IRF4 Toggle-Switch Controls Tolerogenic and Immunogenic Transcriptional Programming in Human Langerhans Cells
Source: Front Immunol. 2021 Jun 15;12:665312. doi: 10.3389/fimmu.2021.665312 (PMC8239435; doi:10.3389/fimmu.2021.665312)
Supplement: Supplementary file 3 [file DataSheet_3.pdf]

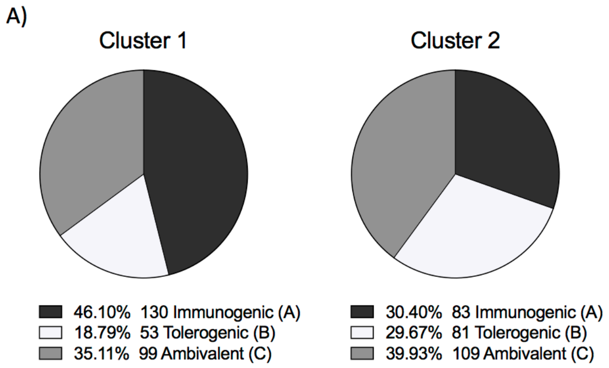

Supplementary figure 3. A toggle switch mathematical model predicts immunogenic vs tolerogenic LC phenotypes from single cell transcriptomic data.

(A) Pie charts summarising the numbers and percentages of cluster 1 and cluster 2 LC assigned to each phenotype through utilising the toggle-switch model for trajectory plotting.
